# Statistical learning of peptide retention behavior in chromatographic separations: a new kernel-based approach for computational proteomics

**DOI:** 10.1186/1471-2105-8-468

**Published:** 2007-11-30

**Authors:** Nico Pfeifer, Andreas Leinenbach, Christian G Huber, Oliver Kohlbacher

**Affiliations:** 1 Division for Simulation of Biological Systems, Center for Bioinformatics, Eberhard-Karls University, 72076 Tübingen, Germany; 2 Department of Chemistry, Instrumental Analysis and Bioanalysis, Saarland University, 66123 Saarbrücken, Germany

## Abstract

**Background:**

High-throughput peptide and protein identification technologies have benefited tremendously from strategies based on tandem mass spectrometry (MS/MS) in combination with database searching algorithms. A major problem with existing methods lies within the significant number of false positive and false negative annotations. So far, standard algorithms for protein identification do not use the information gained from separation processes usually involved in peptide analysis, such as retention time information, which are readily available from chromatographic separation of the sample. Identification can thus be improved by comparing measured retention times to predicted retention times. Current prediction models are derived from a set of measured test analytes but they usually require large amounts of training data.

**Results:**

We introduce a new kernel function which can be applied in combination with support vector machines to a wide range of computational proteomics problems. We show the performance of this new approach by applying it to the prediction of peptide adsorption/elution behavior in strong anion-exchange solid-phase extraction (SAX-SPE) and ion-pair reversed-phase high-performance liquid chromatography (IP-RP-HPLC). Furthermore, the predicted retention times are used to improve spectrum identifications by a *p*-value-based filtering approach. The approach was tested on a number of different datasets and shows excellent performance while requiring only very small training sets (about 40 peptides instead of thousands). Using the retention time predictor in our retention time filter improves the fraction of correctly identified peptide mass spectra significantly.

**Conclusion:**

The proposed kernel function is well-suited for the prediction of chromatographic separation in computational proteomics and requires only a limited amount of training data. The performance of this new method is demonstrated by applying it to peptide retention time prediction in IP-RP-HPLC and prediction of peptide sample fractionation in SAX-SPE. Finally, we incorporate the predicted chromatographic behavior in a *p*-value based filter to improve peptide identifications based on liquid chromatography-tandem mass spectrometry.

## Background

Experimental techniques for determining the composition of highly complex proteomes have been improving rapidly over the past decade. The application of tandem mass spectrometry-based identification routines has resulted in the generation of enormous amounts of data, requiring efficient computational methods for their evaluation. There are numerous database search algorithms for protein identification such as Mascot [1], Sequest [2], OMSSA [3] and X!Tandem [4], as well as de-novo methods like Lutefisk [5] and PepNovo [6]. Furthermore, there are a few methods like InsPecT [7] which use sequence tags for pruning the possible search space using more computationally expensive and more accurate scoring functions afterwards. Database search algorithms generally construct theoretical spectra for a set of possible peptides and try to match these theoretical spectra to the measured ones to find the candidate(s) which match(es) best. In order to distinguish between true and random hits, it is necessary to define a scoring threshold, which eliminates all peptide identifications with scores below the scoring threshold. This threshold value is chosen quite conservatively to get very few false positives. Consequently, there is a significant number of correct identifications below the threshold that are not taken into account, although these spectra often correspond to interesting (e.g. low abundance) proteins. One of the goals of this work was to increase the number of reliable identifications by filtering out false positives in this 'twilight zone', below the typical threshold. There are various studies addressing this issue [8-10] by calculating the probability that an identification is a false positive.

Standard identification algorithms are based on MS/MS data and do not use the information inherent to the separation processes typically used prior to mass spectrometric investigation. Since this additional experimental information can be compared to predicted properties of the peptide hits suggested by MS/MS identification, false positive identifications can be identified. In SAX-SPE, it is important to know whether a peptide binds to the column or flows through. This information can also be incorporated into the identification process to filter out false positive identifications. Oh *et al*. [11] elaborated several chemical features such as molecular mass, charge, length and a so-called sequence index of the peptides. These features were subsequently used in an artificial neural network approach to predict whether a peptide binds to the SAX column or not. The sequence index is a feature reflecting the correlation of pI values of consecutive residues. Strittmater *et al*. [12] included the experimental retention time from an ion-pair reversed-phase liquid chromatographic separation process into a peptide scoring function. They used a retention time predictor based on an artificial neural network [13] but a number of other retention time predictors exist [14,15]. If the deviation between observed and predicted retention time is large, then the score of the scoring function becomes small. Since they only considered the top scoring identifications (rank = 1), they missed correct identifications of spectra where a false positive identification had a larger score than the correct one. We also address these cases in our work, demonstrating that filtering out identifications with a large deviation between observed and predicted retention time significantly improves the classification rate of identifications with small maximal scores. Only recently, Klammer *et al*. [16] used support vector machines (SVMs) [17] to predict peptide retention times. Nevertheless, they used standard kernel functions and stated that they needed at least 200 identified spectra with high scores to train the learning machine.

When applying of machine learning techniques to the prediction of chromatographic retention, a concise and meaningful encoding of the peptide properties is crucial. The features used for this encoding must capture the essential properties of the interaction of the peptide with the stationary and the mobile phases. These properties are mostly determined by the overall amino acid composition, by the sequence of the N-and C-terminal ends, and by the sequence in general. One of the most widely applied machine learning techniques are SVMs. SVMs use a *kernel function *which is used to encode distances between individual data points (in our case, the peptides). There are numerous kernel functions described in the literature which can be applied to sequence data. Some of them are totally position-independent, like the spectrum kernel [18] which basically just compares the frequencies of patterns of a certain length. Other kernels like the locality-improved kernel [19] or the weighted-degree kernel [20] account for patterns at a certain position. Since patterns could occur shifted by a particular amount of characters, the oligo kernel [21] and the weighted-degree kernel with shifts [22] also account for these signals in a range controlled by an additional parameter. All of these kernels (except the spectrum kernel) were introduced for sequences of the same length. However, the length of peptides typically encountered in computational proteomics experiments varies significantly, ranging roughly from 4–40 amino acids. Because it can be assumed that the local-alignment kernel [23], which can also handle sequences of different lengths, does not suit this kind of problem perfectly, we elaborated a new kernel function, which can be applied to sequences of different lengths. Consequently, this new kernel function is applicable to a wide range of computational proteomics experiments.

In 2006 Petritis *et al*. [14] evaluated different features like peptide length, sequence, hydrophobicity, hydrophobic moment and predicted structural arrangements like helix, sheet or coil for the prediction of peptide retention times in reversed-phase liquid chromatography-MS. They used an artificial neural network and showed that the sequence information, together with sequence length and hydrophobic moment yield the best prediction results. In their study, they used only the border residues of the peptide sequences; their evaluation showed that a border length of 25 worked best for their dataset. Since they used one input node for every position of the borders of the peptide, they needed a very large training set, which means that they trained their learning machine on 344,611 peptide sequences.

Since one cannot routinely measure such an amount of training sequences before starting the actual measurements, it is reasonable to apply a sort of gaussian smoothing effect to the sequence positions. This means that in our representation, not every amino acid at every position is considered but rather regions (consecutive sequence positions) where the amino acid occurs. The distance of the amino acids of two sequences is scored with a gaussian function. The size of this region modeled by our kernel function can be controlled by the kernel parameter *σ *of the kernel function and is learned by cross validation. By this and because we use support vector machines in combination with our kernel function, the number of necessary training sequences can be decreased dramatically. By just using the amino acid sequence, we do not rely on features which are important for certain separation processes. This means that we learn the features (i.e. composition (using a large sigma in the kernel function), sequence length, hydrophobic regions ...) which are important for the prediction process within the data because they are reflected in the amino acid sequence. This is why our kernel function can be used for retention time prediction in IP-RP-HPLC as well as for fractionation prediction in SAX-SPE.

When applied to the same dataset as Oh *et al*. [11] used, our kernel function in conjunction with support vector classification predicts 87% of the peptides correctly. This is better than for all reported methods. Furthermore, our retention time prediction model is based on a new kernel function in conjunction with support vector regression [24], which allows us to predict peptide retention times very accurately, requiring only a very small amount of training data. This method has a better performance on a comparative test set than the artificial neural network method used by Strittmater *et al*. [12], even with a much smaller training set. Additionally, our method outperforms the methods introduced by Klammer *et al. *[16]. In the first part of the paper, we demonstrate that our new kernel function, in combination with support vector classification, achieves better results in SAX-SPE fractionation prediction than any published method. Next, we show that our kernel function also performs very well in peptide retention time prediction in IP-RP-HPLC with very few training data required. This allows us to train our predictor on a dataset acquired in one run to predict retention times for two further runs, and to filter the data by deviation in observed and predicted retention time. This leads to a huge improvement in the classification rate of the identifications of spectra for which only identifications with small scores can be found, and also improves the classification rate of high scoring identifications. The "Methods" section briefly gives an introduction to support vector classification and support vector regression. Then our new kernel function is introduced and we explain our *p*-value based filtering approach. Finally, there is an explanation of the datasets used in this study.

## Results and Discussion

In this section, we present the results for two different application areas of our new kernel function. The first one is peptide sample fractionation prediction in SAX-SPE, and the second one is peptide retention time prediction in IP-RP-HPLC experiments. For peptide sample fractionation prediction, we demonstrate that our method performs better than the established method. In retention time prediction, we show that we perform very well with just a fractional amount of training data required. This allows us to train our predictor with a dataset measured in one run to predict retention times of the next runs very accurately. The peptide identifications are improved afterwards by filtering out all peptides which have a large deviation between observed and predicted retention time.

### Performance of Peptide Sample Fractionation Prediction

To be able to compare our results with existing methods, we used the same dataset and the same setup as Oh *et al*. [11]. This means that we randomly partitioned our data into a training set and a test set, having 120 peptides for training and 30 peptides for testing. The performance was measured by classification success rate (SR), which is the number of successful predictions divided by the number of predictions. The whole procedure was repeated 100 times to minimize random effects. The training was conducted by a five-fold cross-validation (CV) and the model was trained using the best parameters from the CV and the whole training set.

To compare our new kernel function with established kernels, we used the best four feature combinations of Oh *et al. *[11] and trained an SVM with the polynomial and the RBF kernel for each feature combination. Feature number one is molecular weight, the second is sequence index, the third is length and the fourth feature is the charge of the peptide. We used the same evaluation setting as described above and in the five-fold CV the SVM parameter *C *∈ {2^-4^·2^*i*^|*i *∈ {0, 2,..., 14}}. For the *σ *parameter of the RBF kernel, *σ *∈ {2^-15^·2^*i*^|*i *∈ {0, 1,..., 24}} and for the degree *d *of the polynomial kernel, *d *∈ {1. 2, 3}. The results are shown in Table 1. It seems as if the fourth feature (i.e. the charge of the peptide) is the most important factor but molecular weight also seems to improve the prediction performance.

**Table 1:** Peptide sample fractionation prediction using standard SVMs. This table shows the classification success rates of the different feature combinations for SVMs with the polynomial and the RBF kernel on the dataset of Oh *et al*. [11]. The features are (1) molecular weight, (2) sequence index, (3) length and (4) charge of the peptide calculated as in [11].

**Feature combination**	**Polynomial kernel**	**RBF kernel**
1, 2, 3, 4	0.78	0.80
1, 2, 3	0.66	0.63
1, 2, 4	0.78	0.80
2, 3, 4	0.75	0.75

**Figure 1:**
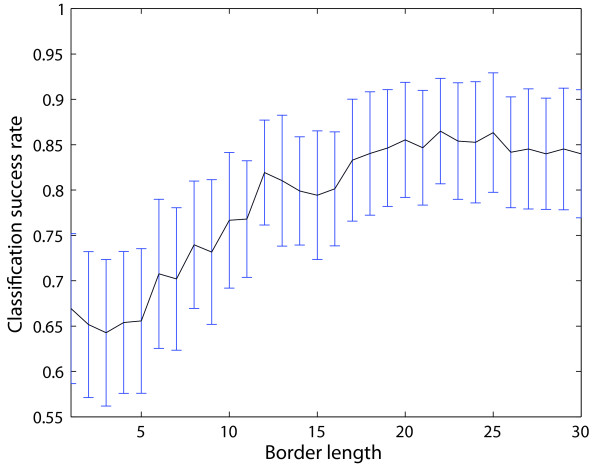
**Border length evaluation of the POBK**. This figure shows the evaluation of SR using different border lengths *b *for the *POBK *on the dataset of Oh *et al*. [11].

An independent approach which just uses the sequence information of the peptides was performed using the local-alignment kernel by Vert *et al*. [23]. Using the same setup as described above, we used the BLOSUM62 matrix [25] and the kernel function parameters were the following: *β *∈ {0.1, 0.2, 0.5, 0.8, 1}, *d *∈ {1, 3, 5, 7, 9, 11, 13} and *e *∈ {1, 3, 5, 7, 9, 11, 13}. Nevertheless, the performance of these kernel approaches led to inferior results than the published method by Oh *et al*. [11]. Therefore more appropriate kernel functions are needed, like our new *paired oligo-border kernel *(*POBK*), which is explained in the "Methods" section. The kernel function has a kernel parameter *b *which is the border length of the peptide. A small *b *means that only few border residues of the peptides contribute to the kernel function, and a border length equal to the sequence length would mean that all residues contribute to the kernel function value. To determine the best border length of the *POBK*, we performed the evaluation for all *b *∈ {1,..., 30}. The evaluation of border length *b *depicted in Fig. 1 shows that for a *b *greater than 19, the SR does not change significantly, with a slight improvement for *b *= 22. This is why in the following, only the *POBK *for *b *= 22 is considered.

**Figure 2:**
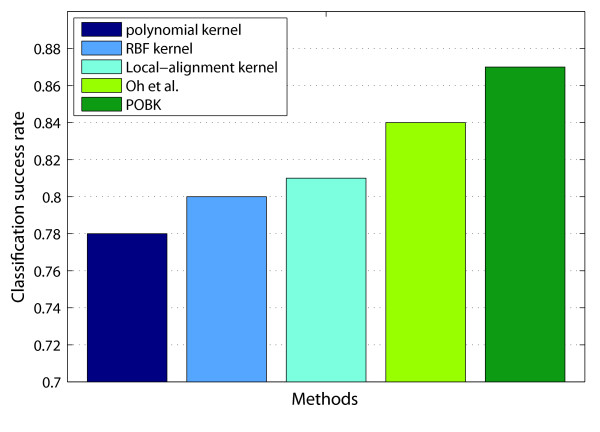
**Performance comparison for peptide sample fractionation prediction**. Comparison of classification success rates for different methods predicting peptide adsorption on the dataset of Oh *et al*. [11].

A comparison of the SR for different methods can be found in Fig. 2. The first two bars represent the SR performance of the best SVMs using standard kernels of Table 1. The third bar demonstrates the performance of an SVM with the local-alignment kernel. The fourth bar shows the performance of the best predictor in Oh *et al*., which is 0.84. The last bar represents the SR of the *POBK*, which is introduced in this paper, for peptide sample fractionation and retention time prediction. The SR of this method is 0.87, which is significantly better than all other approaches.

### Correctly Predicted Peptides in Peptide Sample Fractionation Prediction

In Oh *et al*. [11] the prediction process with 100 random partitionings was done for the best four predictors, and for every peptide, the whole predictions were stored. These authors then classified a peptide by the majority label which had been assigned to the peptide. By this method, they were able to assign 127 of the 150 peptides correctly, which corresponds to an SR of 0.8467.

**Figure 3:**
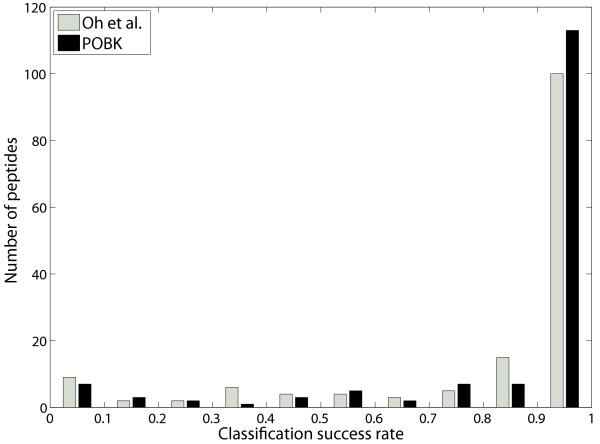
**Histogram of classification success rate**. This figure shows a histogram of the SR of particular peptides using the majority classifier on the dataset of Oh *et al*. [11]. This is compared to the ensemble prediction of Oh *et al*.

To be able to compare this procedure with our method, we made the assumption, that for a particular peptide, the SVM would make a correct assignment more often. Furthermore, we assumed that if we also stored the predictions for each peptide and each run, we could also get a majority predictor which yields good performance. The evaluation of this procedure shows that we are able to predict 134 peptides correctly in this setting, which is an SR of 0.8933. Fig. 3 shows a histogram of the SRs for the different peptides for the method by Oh *et al*. [11] and the SVM with the *POBK*.

### Evaluation of Model Performance for Peptide Retention Time Prediction

For peptide retention time prediction, we had several goals. The first one was to elaborate a retention time predictor showing equivalent performance as established methods but requiring just a fraction of the training set size.

To demonstrate that our retention time predictor fullfills the desired constraints, we performed a two-deep CV on the Petritis dataset [14] described in the "Methods" section. This means that we partitioned the data randomly into ten partitions and performed a CV with the data from nine of the ten partitions to find the best parameters. Later, we trained our model with the best hyperparameters and the data of the nine partitions to evaluate the performance of the predictor on the omitted tenth partition. This was done for every possible combination of the ten partitions and the whole procedure was repeated ten times to minimize random effects.

**Figure 4:**
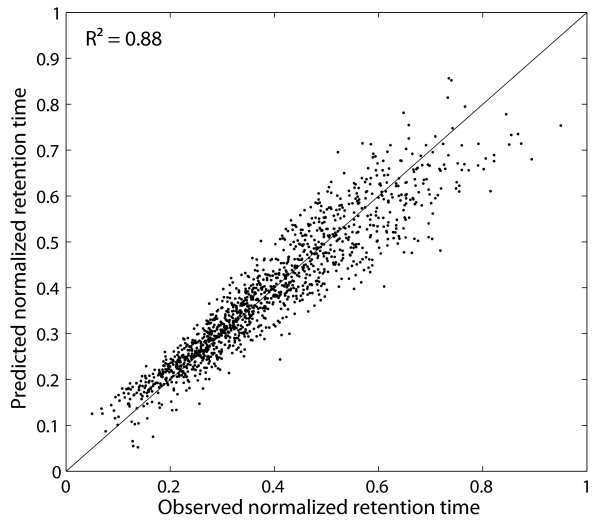
**Example figure for peptide retention time prediction**. This plot shows the observed normalized retention time against the predicted normalized retention time for one of ten two-deep CV runs on the Petritis test set [14]. Since every peptide occurs exactly once in the test set, this plot shows predictions for all of the peptides in the Petritis dataset.

**Figure 5:**
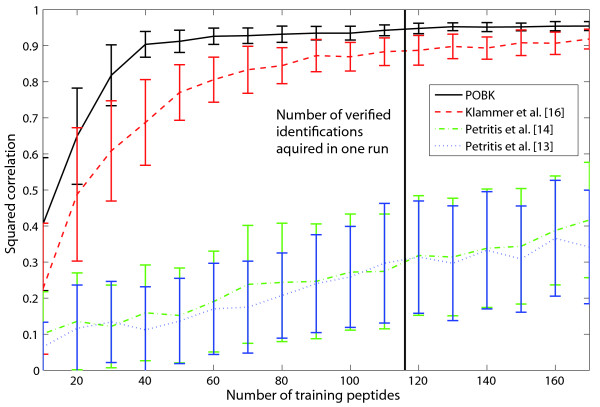
**Learning curve for peptide retention time prediction**. This plot demonstrates the squared correlation coefficient depending on the number of training samples for the union of *vds1 *and *vds2*. For every training sample size, we randomly selected the training peptides, and 40 test peptides and repeated this evaluation 100 times. The plot shows the mean squared correlation coefficients of these 100 runs for every training sample size as well as the standard deviation for the *POBK *and the methods introduced by Klammer *et al. *[16] using the RBF kernel as well as the models by Petritis *et al. *[13, 14]. The vertical line corresponds to the minimal number of distinct peptides in one of our verified datasets which was acquired in one run.

A plot of the observed normalized retention time against the predicted normalized retention time can be seen in Fig. 4 for one of the ten two-deep CV runs. Since the standard deviation over the ten runs was 0.0007, this plot is quite representative for the model performance. Petritis *et al*. [14] showed that their method performs better than those of Meek [26], Mant *et al. *[27], Krokhin *et al*. [28] and Kaliszan *et al*. [29], using this dataset for validation. Thus, in Table 2, we only compare the performance of our method with the work of Petritis *et al*. [14]. This comparison is somewhat biased since we only had a fraction of the original validation set for training, which means that our training set size was 300 times smaller than that of the other methods. Nevertheless, our method performs better than the model [13] which is used by Strittmater *et al*. [12] in their filtering approach. The only model with a better performance is the artificial neural network with 1052 input nodes and 24 hidden nodes [14]. It is obvious that a model like this needs a very large amount training data. Petritis *et al. *[14] trained their model with more than 344,000 training peptides. Therefore, this type of model is not suitable for retention time prediction for measurements under different conditions or with different machines because it is very time consuming to acquire identification and retention time data for more than 344,000 training peptides before starting the actual measurements. To demonstrate that our method is robust enough for training on verified data of one single run, we constructed a non-redundant dataset out of datasets *vds1 *(available as Additional file 1) and *vds2 *(available as Additional file 2). A detailed description of these datasets can be found in the "Methods" section. For different training sizes *s *∈ {10, 20,..., 170}, we randomly selected s peptides for training and 40 peptides for testing. Fig. 5 indicates that for the *POBK*, 40 verified peptides are enough to train a predictor which has a squared correlation coefficient between observed and predicted normalized retention time greater than 0.9 on the test set. This number is much smaller than the number of verified peptides we get for one run since *vds1 *has 144 peptides, *vds2 *has 133 peptides and *vds3 *(available as Additional file 3) has 116. This evaluation shows that with our predictor, it is possible to measure one calibration run with a well defined and easily accessible peptide mixture prepared from real biological samples to train a predictor, which can then be used to predict retention times for the peptides very accurately. Furthermore, Fig. 5 shows a comparison of the *POBK *to the methods introduced by Klammer *et al*. [16] and Petritis *et al*. [13,14] as described in the "Methods" section. Our method needs significantly less training data for a good prediction and has also superior performance if all training sequences of our dataset are used. One possible explanation for the low performance of the models from Petritis *et al*. is that their models need a larger amount of training data. This is supported by the fact that they used about 7000 [13] and about 345,000 [14] training peptides in their studies. To compare our method with the work by Krokhin [30], we used our verified datasets. This means that we e.g. trained our model on *vds1 *and predicted the retention times for peptides of the union of *vds2 *and *vds3*, which were not present in *vds1*. This means that if a peptide occured in *vds2 *and in *vds3*, we only kept the peptide identification with the biggest score. For the *POBK*, we performed a five-fold CV with SVM parameters *C *∈ {2^*i*^|*i *∈ {-9, -8,..., 0}}, *v *∈ {0.4·1.2^*i*^|*i *∈ {0, 1, 2}} and *σ *∈ {0.2·1.221055^*i*^|*i *∈ {0, 1,..., 21}} to determine the best parameters.

**Table 2:** Comparison of different retention time predictors. This table shows the squared correlation coefficient between observed and predicted normalized retention time of retention time prediction methods of Petritis *et al. *[13, 14] on the Petritis test set [14]. These values are compared to our method, the *POBK*, on the Petritis test set [14]. The second column gives the number of training sequences used. For the last two rows, subsets of the data were chosen randomly so that 100 respectively 200 training peptides were selected.

**Method**	**Number of training sequences**	**Squared correlation coefficient**
Petritis *et al*. 2003 [13]	344,611	0.870
Petritis *et al*. 2006 [14]	344,611	0.967
**This work**	**1040**	**0.880**
	**200**	**0.854**
	**100**	**0.805**

Afterwards we trained our model with the whole training set and the best parameters and measured the squared correlation between observed and predicted retention time on the test set. This procedure was repeated ten times to minimize random effects. Since there exists a web-server for the method by Krokhin [30], we could also compare the observed retention times with the predicted ones on our test sets with this method. To calculate the hydrophobicity parameters *a *and *b *of this method, we used our two standard peptides introduced in the "Methods" section. Furthermore, we used the 300 Å column since the other coloumns lead to inferior results. As can be seen in Table 3, the model by Krokhin performs quite well even though it had been elaborated on another type of sorbent. Nevertheless the *POBK *achieves a significantly higher squared correlation coefficient. It should be noted that the web-server by Krokhin is restricted to three different coloumns. The advantage of our method is that there is not any restriction to a certain type of experimental setup. One only needs a small amount of training peptides and can train a model which can immediately be used for retention time prediction. It should be mentioned that the *POBK *has a higher squared correlation between observed and predicted retention time on our datasets than on the testset by Petritis *et al*. This could be due to the fact that Petritis *et al*. performed shotgun proteomics peptide identification [14]. It is commonly accepted that shotgun proteomics peptide identification has a significant false positive rate.

**Table 3:** Evaluation of prediction performance for retention time prediction using the POBK. This table shows the performances of the *POBK *using our verified datasets (introduced in the "Methods" section). The other columns contain the squared correlation coefficient between the observed normalized retention times and the predicted ones for the *POBK *and the method by Krokhin [30].

**Training set**	**Test set**	**POBK**	**Krokhin [30]**
*vds1*	(*vds2 *∪ *vds3*)\*vds1*	0.9570	0.9101
*vds2*	(*vds1 *∪ *vds3*)\*vds2*	0.9564	0.9212
*vds3*	(*vds1 *∪ *vds2*)\*vds3*	0.9521	0.9229

### Improving Peptide Identifications by Using Retention Time Prediction

The second goal for retention time prediction was to elaborate a retention time filter which could be used for improving peptide identifications. In this setting, we trained our learning machine on one of the *vds *(i.e. *vds1*) and predicted the retention times for the remaining *ds *(i.e. *ds2 *and *ds3*). The peptides of the training and test sets were made disjoint by removing all identifications of the test set which belonged to spectra having an identification which was also present in the training set. On every training set, we performed a five-fold CV with SVM parameters *C *∈ {2^*i*^|*i *∈ {-9, -8,..., 0}}, *v *∈ {0.4·1.2^*i*^|*i *∈ {0, 1, 2}} and *σ *∈ {0.2·1.221055^*i*^|*i *∈ {0, 1,..., 21}}. Since the results of the *POBK *for all three datasets in Table 3 show nearly the same very good squared correlation coefficient of about 0.95 between observed and predicted normalized retention times, we restricted ourselves in the following to training our learning machine on *vds3 *and evaluated the filtering capability of our filtering approach on *ds1 *and *ds2*.

The performance evaluation of our filter model was done by a two-step approach. In the first step, we measured the number of true positives and the number of false positives for the identifications returned by the Mascot [1] search engine. This was conducted for different significance values. Mascot provides a significance threshold score for the peptide identification at a given significance level. This significance level was 0.05 in all our studies. To be able to compare the identification performance for different levels of certainty we chose different fractions of the significance threshold score. This means for example, that for a fraction of 0.5, all identifications have to have a score which is equal to or greater than half of the significance threshold score. The evaluation was accomplished for varying threshold fractions *t *∈ {0.01, 0.02,..., 1}. In this setting, we could evaluate the classification rate (CR). This is the number of true identifications divided by the number of spectra having at least one identification with a score higher than t times the significance threshold score. If there was more than one identification with the maximal score for one spectrum, the spectrum was excluded from the evaluation. In the second step, we filtered the data by our retention time model which was learnt on the training set and conducted the same evaluation as in the first step. After this we compared the classification performance of these two evaluations.

**Figure 6:**
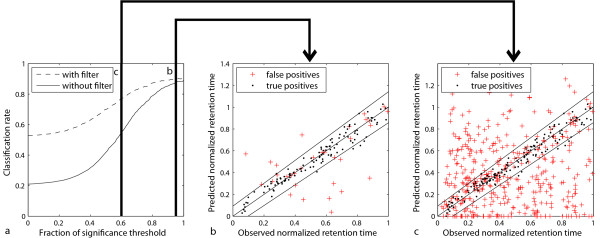
**Visualization of filter performance**. This plot shows the improvement in classification rate one can get by using our retention time filter for a) varying fractions of the significance threshold value, b) all predictions of spectra having a score equal or greater than 95% of the significance threshold value, c) all predictions of spectra having a score equal or greater than 60% of the significance threshold value. The model was trained using the *vds3 *dataset and the performance was measured on *ds1 *and *ds2*. If there was more than one spectrum with the same identification we plotted the mean values of the observed NRTs against the predicted NRT.

Fig. 6a demonstrates the good CR for identifications with high Mascot scores since a threshold fraction equal to one means that all identifications have a score equal or larger than the significance threshold score given by the Mascot search engine. Nevertheless, even for these identifications, filtering with the retention time filter improves the CR from 89–90%. An even greater improvement can be achieved for identifications with smaller scores. If all identifications are constrained to have a score equal or larger than 60% of the significance threshold score, the CR improves from 55–77% by using our filter. A CR of 0.77 is still quite good and, as can be seen in Table 4, the number of true positives increases from 350 to 557. This means that many more spectra can be identified with an acceptable number of false positives by applying our retention time filtering approach. Fig. 6b shows that our model is valuable for removing false identifications since many false positives are outside the trapezoid and are removed by our filter for a threshold fraction of 0.95. Figure 6c shows this even more drastically for a threshold fraction of 0.6. The whole evaluation shows that our retention time prediction can be used to improve the level of certainty for high-scoring identifications and also to allow smaller thresholds to find new identifications with an acceptable number of false positives.

**Table 4:** Evaluation of filter performance. This table presents the classification rates of the identified spectra for varying fractions of the significance threshold with and without retention time filtering. The model was trained using the *vds3 *dataset and the performance was measured on *ds1 *and *ds2*. In this context, tp stands for the number of true positives and fp for the number of false positives. The CR is tp divided by the sum of tp and fp.

**Fraction of threshold**	**tp**	**fp**	**CR**	**tp with filter**	**fp with filter**	**CR with filter**
0.0	683	2572	0.2098	699	626	0.5275
0.1	682	2460	0.2171	692	602	0.5348
0.2	678	2260	0.2308	683	555	0.5517
0.3	669	1909	0.2595	668	483	0.5804
0.4	654	1410	0.3169	646	380	0.6296
0.5	624	868	0.4182	609	261	0.7000
0.6	575	474	0.5481	557	166	0.7704
0.7	516	235	0.6871	500	103	0.8292
0.8	468	125	0.7892	452	66	0.8726
0.9	420	72	0.8537	404	49	0.8918
1.0	366	46	0.8883	350	38	0.9021

## Conclusion

In this paper, we introduced a new kernel function which was successfully applied to two problems in computational proteomics, namely peptide sample fractionation by SAX-SPE and high resolution peptide separation by IP-RP-HPLC. Furthermore, we demonstrated that the predicted retention times can be used to build a *p*-value based model which is capable of filtering out false identifications very accurately.

Our method performs better than all previously reported peptide sample fractionation prediction methods and for retention time prediction, our method is (to our knowledge) the only learning method which can be trained with a small training size of 40 peptides but still achieving a high correlation between observed and predicted retention times. This small required training set allows us to imagine the following application which would be very helpful for proteomic experiments. One could identify a well defined protein mixture before starting the experiments and use the verified peptides for training the predictor. Next the predictor can be used to predict retention times for all identifications of the following runs. This predicted retention time can then be applied to improve the certainty of the predictions. It can also be used to identify a much larger number of spectra with an acceptable number of false positives. This is achieved by lowering the significance threshold and filtering the identifications by our *p*-value-based retention time filter. Since all our methods are integrated into the OpenMS [31] library, which is open source, every researcher is able to use the presented methods free of charge. Also, we offer the prediction models as tools which are part of the OpenMS proteomics pipeline (TOPP) [32]. These tools can be easily combined with other tools from TOPP, allowing wide-range research applications in computational proteomics.

## Methods

### Algorithmical Methods

In this work, we introduce a new kernel function which can be used to predict peptide properties using support vector classification and *v*-support vector regression (*v*-SVR) [24]. We apply this kernel function to predict fractionation of peptides in SAX-SPE as well as peptide retention times in IP-RP-HPLC. To show the superior performance of the new kernel function, we provide comparisons to established kernel functions and the latest approaches of other working groups [11,14,16].

### Support Vector Machines

In binary classification, the task is to find a function *f*: X → Y, Y = {-1, 1} from *n *labelled training samples (*x*_*i*_, *y*_*i*_) ∈ {(*x*_*i*_, *y*_*i*_)|*x*_*i *_∈ X, *y*_*i *_∈ Y, *i *= 1,..., *n*}, such that unlabelled data samples *x *∈ X from the same data source can be classified by this function. The idea is to learn something about the distribution of the training samples so that unseen test examples that belong to the same underlying distribution can be predicted very accurately by the function. In support vector classification [17], the task is to find a discriminating hyperplane in a certain space. Therefore, one normally maximizes

$$\sum_{i}\alpha_{i}-\frac{1}{2}\sum_{i,j}\alpha_{i}\alpha_{j}y_{i}y_{j}\langle x_{i},x_{j}\rangle$$

subject to:

$$\begin{array}{l} 0\leq \alpha_{i}\leq C,\\ \sum_{i}\alpha_{i}y_{i}=0 \end{array}$$

The *C *is chosen beforehand and the optimal weights *α*_*i *_are searched. With the *α*_*i*_s the discriminant function is:

$$f(x)=\text{sign}(\sum_{i}\alpha_{i}\langle x_{i},x\rangle )$$

To be able to learn non-linear discriminant functions it is possible to apply a mapping function to the input variables Φ: X → ℱ as stated in [24]. Since computing the inner product ⟨Φ(*x*_*i*_), Φ(*x*_*j*_)⟩ of the mapped feature vectors in feature space can be very time-expensive, a kernel function *k *can be used instead *k*: X^2 ^→ Y: *k*(*x*_*i*_, *x*_*j*_) = ⟨Φ(*x*_*i*_), Φ(*x*_*j*_)⟩, which implicitly computes the results of the inner product in feature space. The corresponding kernel matrix has to be positive semi-definite. Consequently, the classification function is learnt by maximizing

$$\sum_{i}\alpha_{i}-\frac{1}{2}\sum_{i,j}\alpha_{i}\alpha_{j}y_{i}y_{j}k(x_{i},x_{j})$$

and the discriminant is

$$f(x)=\text{sign}(\sum_{i}\alpha_{i}k(x_{i},x))$$

and the *x*_*i*_: *α*_*i *_> 0 are called *support vectors*.

### Support Vector Regression

In regression, the task is to find a function *f*: X → Y, Y ⊆ ℝ from *n *labelled training samples (*x*_*i*_, *y*_*i*_) ∈ {(*x*_*i*_, *y*_*i*_)|*x*_*i *_∈ X, *y*_*i *_∈ Y, *i *= 1,..., *n*} such that unlabelled data samples *x *∈ X from the same data source can be assigned a label *y *∈ Y by this function. The idea is, as in the binary case, to learn something about the distribution of the training samples so that unseen test examples which belong to the same underlying distribution can be predicted very accurately by the function. In *v*-SVR [24], the regression function is learnt by maximizing

$$W(\alpha^{\left(*\right)})=\sum_{i=1}^{l}(\alpha_{i}^{*}-\alpha_{i})y_{i}-\frac{1}{2}\sum_{i,j=1}^{l}(\alpha_{i}^{*}-\alpha_{i})(\alpha_{j}^{*}-\alpha_{j})\langle x_{i},x_{j}\rangle$$

subject to:

$$\begin{array}{c} \sum_{i=1}^{l}(\alpha_{i}-\alpha_{i}^{*})=0\\ \alpha_{i}^{*}\in \left[0,\frac{C}{l}\right]\\ \sum_{i=1}^{l}(\alpha_{i}+\alpha_{i}^{*})\leq C\cdot \nu \end{array}$$

In this term, the *v *bounds the amount of training errors and support vectors of the function. To be able to learn non-linear discrimination functions, it is again possible to apply a mapping function to the input variables Φ: X → ℱ and a kernel function which corresponds to the inner product of the mapped feature vectors. Consequently, the regression function is learnt by maximizing

$$W(\alpha^{\left(*\right)})=\sum_{i=1}^{l}(\alpha_{i}^{*}-\alpha_{i})y_{i}-\frac{1}{2}\sum_{i,j=1}^{l}\left(\alpha_{i}^{*}-\alpha_{i})(\alpha_{j}^{*}-\alpha_{j})k(x_{i},x_{j}\right)$$

### Kernel Function

The oligo kernel introduced by Meinicke *et al*. in [21] is a kernel function which can be used to find signals in sequences for which the degree of positional uncertainty can be controlled by the factor *σ *of the kernel function. The standard oligo kernel was introduced for sequences of fixed length. Since there are many problems like peptide retention time prediction in which the length of the sequences varies significantly, this kernel function cannot be applied to them directly.

Petritis *et al*. [14] predicted peptide retention times very accurately by encoding the border residues directly. As stated in [33], the oligo kernel can be used as a motif kernel. This motivated us to construct a kernel which only considers the border residues of a peptide for a fixed border length *b*. Consequently, the kernel function is called *oligo-border kernel *(*OBK*). Here, a motif is a certain *k*-mer at a position inside the *b *residue border at each side where *b *∈ {1,..., 30}. This means that every *k*-mer at the leftmost *b *residues contributes to its oligo function as well as every *k*-mer at the rightmost *b *ones. For the peptide sequence *s *∈ A^*n*^, the left border *L *is defined as *L *= {1, 2,..., min(*n*, *b*)} and *R *= {max(0, *n *- *b *+ 1),..., *n*}. The set $S_{\omega }^{L}$ = {*p*_1_, *p*_2_,...} contains the positions where the *k*-mer *ω *∈ A^*k *^occurs inside the left border and $S_{\omega }^{R}$ ={*p*_1_, *p*_2_,...} the *k*-mer positions for the right border. This means that $S_{\omega }^{L}$ ∩ *L *= $S_{\omega }^{L}$ and $S_{\omega }^{R}$ ∩ *R *= $S_{\omega }^{R}$. In [21] the feature space representation of a sequence is a vector containing all of its oligo functions. These oligo functions are the sums of gaussians for every particular *k*-mer. This means that

$$\mu_{\omega }(t)=\sum_{p\in S_{\omega }}\exp(-\frac{1}{2\sigma^{2}}{\left(t-p\right)}^{2})$$

Consequently, the *oligo-border *function is:

$$\mu_{\omega }^{M}(t)=\sum_{p\in S_{\omega }^{M}}\exp(-\frac{1}{2\sigma^{2}}{\left(t-p\right)}^{2})$$

where *M *∈ {*L*, *R*}. This leads directly to the feature map:

$$\Phi (s)={\left[\mu_{\omega_{1}}^{L}(t),...,\mu_{\omega_{\left|A^{k}\right|}}^{L}(t),\mu_{\omega_{1}}^{R}(t),...,\mu_{\omega_{\left|A^{k}\right|}}^{R}(t)\right]}^{T}$$

Let *U *= *L *∪ *R *and let $S_{\omega }^{U_{i}}$ be the set $S_{\omega }^{U}$ of sequence *s*_*i*_. Let

ind(*p*, *q*) = [[(*p *∈ *L*_*i *_∧ *q *∈ *L*_*j*_)|(*p *∈ *R*_*i *_∧ *q *∈ *R*_*j*_)]]

for *p *∈ *U*_*i *_and *q *∈ *U*_*j *_in which [[*condition*]] is the indicator function. This function equals one if *condition *is true and zero otherwise. Similar to [21], the kernel function is then

$$k_{OBK}(s_{i},s_{j})=\sqrt{\pi }\sigma \sum_{\omega \in A^{k}}\sum_{p\in S_{\omega }^{U_{i}}}\sum_{q\in S_{\omega }^{U_{j}}}\text{ind}(p,q)\cdot e^{-\frac{{\left(p-q\right)}^{2}}{4\sigma^{2}}}$$

A further variant of the *OBK *is to consider similarities between opposite borders. This means that there is only one oligo function for a certain oligo and the occurrence positions of signals in the right border are numbered from one to min(*n*, *b*) from right to left. In this way, a high similarity between the right border of a peptide and the left border of another peptide can also be detected. Throughout the paper, this kernel is called the *paired oligo-border kernel *(*POBK*) and the kernel function is:

$$\begin{array}{ccc} k_{POBK}(s_{i},s_{j}) & = & \sqrt{\pi }\sigma \sum_{\omega \in A^{k}}\\ & \times & [\sum_{p\in S_{\omega }^{U_{i}}}\sum_{q\in S_{\omega }^{U_{j}}}\text{ind}(p,q)\cdot e^{-\frac{{\left(p-q\right)}^{2}}{4\sigma^{2}}}\\ & + & \sum_{p\in S_{\omega }^{R_{i}}}\sum_{q\in S_{\omega }^{L_{j}}}e^{-\frac{{\left((n-p+1)-q\right)}^{2}}{4\sigma^{2}}}\\ & + & \sum_{p\in S_{\omega }^{L_{i}}}\sum_{q\in S_{\omega }^{R_{j}}}e^{-\frac{{\left(p-(n-q+1)\right)}^{2}}{4\sigma^{2}}}] \end{array}$$

This kernel function can be computed as efficiently as the oligo kernel by appropriate position encoding. The kernel matrix is positive definite which follows directly from [33]. Since preliminary experiments showed that the *POBK *works better than the *OBK*, we used only the *POBK *in this study. Furthermore, the preliminary experiments showed that the best performance of the *k*-mer length is one which is quite reasonable, since the peptides are very short compared to the number of different amino acids. This is also supported by the study [34] on protein sequences, in which histograms of monomer distances performed better than distance histograms of longer *k*-mers. A combination of different lengths as in [33] also led to inferior results, which could be due to the normalization of the single kernel functions. Consequently, in this study, we only used *k*-mer length one.

### *P*-value Calculation and Filtering

As stated earlier, the retention time prediction is used in this work to improve the certainty of peptide identifications found by search engines like Mascot and to filter out false identifications. This is done by fitting a linear model to the prediction data in the training set. The model reflects the fact that retention times of late eluting peptides show a higher deviation than early ones. The poorer performance in retention time prediction for longer peptides was also observed in [14] supporting this fact. For our predictions, we therefore match an area to the prediction data of the training set which contains ≥95% of the points and is the wider the bigger the corresponding retention time is. An application of the model can be found in Fig. 6b and Fig. 6c. We call the smallest distance in the model *γ*_0 _at normalized retention time (NRT) equal to zero, and *γ*_*max *_is the biggest gamma at NRT = 1. We can consequently calculate a corresponding gamma for every normalized retention time *t*_*nor *_by *γ *= *γ*_0 _+ *t*_*nor *_·(*γ*_*max *_-*γ*_0_). Since we assume gaussian error distribution gamma corresponds to 2·*standard deviation *of the normal distribution such that a *p*-value can be calculated for every retention time prediction by calculating the probability that a correct identification has a bigger deviation between observed and predicted normalized retention time. The null hypothesis is that the identification is correct. For filtering identifications, we use these *p*-values in the following way.

Since we do not want to filter out correct identifications, the probability of filtering out a correct identification can be controlled by a significance level. In the experiments, we set the significance level to 0.05. This means that the probability that a correct identification has a deviation between observed and predicted retention time equal or greater than the allowed deviation is 0.05. Consequently, the probability of filtering out correct identifications is 0.05. Concerning the *p*-values mentioned above, this means that *p *has to be bigger than 0.05. Basically, for significance level 0.05, this means that every identification outside the fitted model is filtered out and the identifications inside are kept.

### Computational Resources

All methods elaborated in this work were integrated by us into OpenMS, a software platform for shotgun proteomics [31] which has a wrapper for the libsvm [35]. This library was used for the support vector learning. Furthermore, we integrated the prediction models into TOPP [32]. Some additional evaluations for peptide sample fractionation prediction were performed using shogun [36].

### Experimental Methods and Additional Data Sets

For peptide sample fractionation prediction, we used the data from Oh *et al*. [11] to show the superior performance of our method. For peptide retention time prediction, we used different datasets. The first one is a validation dataset which was used by Petritis *et al*. in 2006 [14] to predict peptide retention times using artificial neural networks. In their experiment, they measured more than 345,000 peptides, and chose 1303 high confident identifications for testing and the remaining peptides for training. Since they only published the 1303 test peptides, we could only use this small number of peptides. The dataset was used in our study to be able to show the performance of our methods compared to other well established methods for peptide retention time prediction. Further datasets for retention time prediction were measured in our labs to show that training on the data of one run suffices to predict retention times on the next runs very accurately and to improve spectrum identifications significantly.

### Experimental Setup

The datasets for training and evaluation of the retention time predictor had to fulfill two basic requirements. First, the identity of the studied peptides had to be known with high certainty in order to avoid incorrect sequence annotations for the training dataset, and second, retention times had to be measured with high reproducibility. Altogether, we measured 19 different proteins, which were purchased from Sigma (St. Louis, MO) or Fluka (Buchs, Switzerland). To avoid excessive overlapping of peptides in the chromatographic separations, the proteins were divided into three artificial protein mixtures and subsequently digested using trypsin (Promega, Madison, WI) using published protocols [37]. The protein mixtures contained the following proteins in concentrations between 0.4 – 3.2 pmol/*μ*l:

Mixture 1: *β*-casein (bovine milk), conalbumin (chicken egg white), myelin basic protein (bovine), hemoglobin (human), leptin (human), creatine phosphokinase (rabbit muscle), *α*1-acid-glycoprotein (human plasma), albumin (bovine serum).

Mixture 2: cytochrome C (bovine heart), *β*-lactoglobulin A (bovine), carbonic anhydrase (bovine erythrocytes), catalase (bovine liver), myoglobin (horse heart), lysozyme (chicken egg white), ribonuclease A (bovine pancreas), transferrin (bovine), *α*-lactalbumin (bovine), albumin (bovine serum).

Mixture 3: thyroglobulin (bovine thyroid) and albumin (bovine serum).

Adding albumin to each protein mixture was performed because in each run, there had to be an identical set of peptides to normalize the retention times. The resulting peptide mixtures were then separated using capillary IP-RP-HPLC and subsequently identified by electrospray ionization mass spectrometry (ESI-MS) as described in detail in [37,38]. The separations were carried out in a capillary/nano HPLC system (Model Ultimate 3000, Dionex Benelux, Amsterdam, The Netherlands) using a 50 × 0.2 mm monolithic poly-(styrene/divinylbenzene) column (Dionex Benelux) and a gradient of 0–40% acetonitrile in 0.05% (v/v) aqueous trifluoroacetic acid in 60 min at 55°C. The injection volume was 1 *μ*l, and each digest was analyzed in triplicate at a flow rate of 2 *μ*l/min. On-line ESI-MS detection was carried out with a quadrupole ion-trap mass spectrometer (Model esquire HCT, Bruker Daltonics, Bremen, Germany).

### Identification of Spectra

Peptides were identified on the basis of their tandem mass spectra (maximum allowed mass deviations: precursor ions: ± 1.3 Da, fragment ions: ± 0.3 Da) using Mascot [1] (version 2.1.03). The database was the Mass Spectrometry Database, MSDB (version 2005-02-27) restricted to chordata (vertebrates and relatives). We allowed one missed cleavage as well as charges 1+, 2+ and 3+. The mass values were monoisotopic. The significance level of the significance threshold score for the peptide hits was 0.05. Since the amino acid sequences of the 19 proteins of our mixtures are known, we could verify the identifications by sequence comparison with the protein sequences. To avoid random verifications, we restricted the peptide length to be equal or greater than six. The whole process led to two datasets for each protein mixture – one which only contained the verified peptides and the other one with all Mascot identifications. In this paper, we call the datasets containing the verified peptide sequences *vds *and the datasets with all Mascot identifications *ds*. The *vds*s are used to train the predictors and the *ds*s are used to access the classification performance of the identification process.

### Normalization of Retention Times

We chose two standard peptides which were identified in all of the runs. One of these peptides, which had the amino acid sequence TCVADESHAGCEK, eluted very early and the other one, which had the amino acid sequence MPCTEDYLSLILNR, eluted very late. We scaled the retention times linearly so that the early eluting peptide got an NRT of 0.1 and the late eluting peptide an NRT of 0.9. All peptides with an NRT below zero or above 1 were removed. The lists of identified peptides of *vds1*, *vds2 *and *vds3*, together with their respective retention times, are available as Additional files 1, 2 and 3 in the supplementary material.

### Reimplementation of Existing Methods for Comparison Purposes

For retention time prediction we compared our method with several methods. Therefore we had to reimplement the methods by Klammer *et al*. [16] as well as the methods by Petritis *et al. *[14]. For the methods by Klammer *et al*., we implemented the same encoding as described in the literature and used the RBF kernel of the libsvm [35]. The cross validation was performed with the same parameter ranges as described in the paper (*C *∈ {10 ^-3^, 10^-2^,..., 10^7^} and *σ *∈ {10^-6^, 10^-7^, 10^-8^}). For comparison with the models by Petritis *et al*. we reimplemented the models as described in the literature using Matlab R2007a (The MathWorks, Inc., United States) and the neural networks toolbox version 5.0.2 (The MathWorks, Inc.). This means that for the first model of Petritis *et al*. [13] we had a feedforward neural network with 20 input nodes, two hidden nodes and one output node. The frequencies of the amino acids of the peptides served as input. For the second model of Petritis *et al*. [14] we had 1052 input nodes, 24 hidden nodes and one output node. The amino acids at the 25 leftmost and the 25 rightmost residues served as input as well as the length and the hydrophobic moment of the peptide as described in [14]. Both models were trained using a backpropagation algorithm.

## Authors' contributions

OK and CH designed the experiment and the study. AL was responsible for the experimental data generation. NP developed and implemented the theoretical methods and performed the data evaluation. All authors contributed to the writing of the manuscript.

## Supplementary Material

Additional file 1 **Verified data set one (vds1)**. vds1.csv lists the identified peptides of *vds1 *with normalized retention time, observed retention time, precursor mass, charge, score and significance threshold score (at significance level *p *= 0.05). Click here for file

Additional file 2 **Verified data set two (vds2)**. vds2.csv lists the identified peptides of *vds2 *with normalized retention time, observed retention time, precursor mass, charge, score and significance threshold score (at significance level *p *= 0.05). Click here for file

Additional file 3 **Verified data set three (vds3)**. vds3.csv lists the identified peptides of *vds3 *with normalized retention time, observed retention time, precursor mass, charge, score and significance threshold score (at significance level *p *= 0.05). Click here for file
